# Reduction of exogenous ketones depends upon NADPH generated photosynthetically in cells of the cyanobacterium *Synechococcus *PCC 7942

**DOI:** 10.1186/2191-0855-1-24

**Published:** 2011-09-01

**Authors:** Rio Yamanaka, Kaoru Nakamura, Akio Murakami

**Affiliations:** 1 Faculty of Pharmaceutical Science, Himeji-Dokkyo University, 7-2-1 Kami-Ohno, Himeji, Hyogo 670-8524, Japan; 2 Graduate School of Human Development and Environment, Kobe University, 3-11 Tsurukabuto, Nada-ku, Kobe, Hyogo 657-8501, Japan; 3 Kobe University Research Center for Inland Seas, 2746 Iwaya, Awaji, Hyogo 656-2401, Japan

**Keywords:** biocatalysis, cyanobacteria, ketone reduction, light energy, NADPH, photosynthesis

## Abstract

Effective utilization of photosynthetic microorganisms as potential biocatalysts is favorable for the production of useful biomaterials and the reduction of atmospheric CO_2_. For example, biocatalytic transformations are used in the synthesis of optically active alcohols. We previously found that ketone reduction in cells of the cyanobacterium *Synechococcus *PCC 7942 is highly enantioselective and remarkably enhanced under light illumination. In this study, the mechanism of light-enhanced ketone reduction was investigated in detail using several inhibitors of photosynthetic electron transport and of enzymes of the Calvin cycle. It is demonstrated that light intensity and photosynthesis inhibitors significantly affect the ketone reduction activity in *Synechococcus*. This indicates that the reduction correlates well with photosynthetic activity. Moreover, ketone reduction in *Synechococcus *specifically depends upon NADPH and not NADH. These results also suggest that cyanobacteria have the potential to be utilized as biocatalytic systems for direct usage of light energy in various applications such as syntheses of useful compounds and remediation of environmental pollutants.

## Introduction

Most chlorophyll-containing organisms are photoautotrophic and manufacture various organic compounds from inorganic carbon in the form of CO_2 _using solar light energy. All other organisms except for chemosynthetic bacteria are heterotrophic and ultimately depend upon photoautotrophic organisms to provide their energy and nutrients (Anemaet et al. 2010). Oxygen-evolving photoautotrophic organisms, plants, algae, and cyanobacteria have significantly influenced the global environment and carbon cycle on Earth. Improved utilization of such photoautotrophic organisms will be essential in preventing acceleration of a rise in anthropogenic CO_2_, which is believed to cause global warming and ocean acidification (Zabochnicka-świątek 2010). In particular, aquatic microalgae and cyanobacteria are among the most promising candidates in efforts to reduce atmospheric CO_2 _because they have higher photosynthetic activity and proliferate faster than terrestrial plants (Kurano et al. 1998). For this purpose, large-scale mass-culture of microalgae and cyanobacteria has been accomplished using either outdoor open pond processes or enclosed bioreactor systems (Takano et al. 1992, Ugwu et al. 2005, Kumar et al. 2010).

Optically active alcohols are useful for organic syntheses of chemical catalysts, liquid crystals, flavors, agrochemicals, and drugs (Goldberg et al. 2007a, b, Moore et al. 2007, Huisman et al. 2010). There have been many reports on the synthesis of optically active alcohols using chemical and biological catalysts (Nakamura et al. 2003, Carey et al. 2006, Matsuda et al. 2009). Most of the reports on biocatalytic transformations have been focused on non-photosynthetic and heterotrophic microorganisms or their isolated enzymes (Nakamura et al. 2003, Matsuda et al. 2009). In these biocatalytic reactions, organic compounds, such as sugars produced by photosynthetic organisms, are used as crucial energy sources. These biocatalytic reactions indirectly and ultimately depend upon solar light energy. In terms of energy acquisition efficiency, photoautotrophic organisms appear to be the most effective biocatalytic agents (Nakamura 2007). In fact, there have been several reports on asymmetric reduction using photoautotrophs such as microalgae (Noma and Asakawa 1992, Kuramoto et al. 1999, Nakamura et al. 2000, Utsukihara et al. 2004, Shimoda et al. 2004, Itoh et al. 2005, Utsukihara et al. 2006, Takemura et al. 2009), plant cultured-cells (Kojima et al. 2009), and germinated plants (Matsuo et al. 2008, Takeda et al. 2011). Enzymatic systems isolated from a microalga were used in combination with NADPH (nicotinamide adenine dinucleotide phosphate) to perform a biotransformation such as biocatalytic reduction (Shimoda and Hirata 2000).

To develop the use of autotrophic biocatalysts for reduction of exogenous substances, mechanistic studies on biocatalytic reduction reactions are necessary. Particularly, the effect of light on the reaction should be investigated in detail because light is needed in order to take advantage of the catalytic power of autotrophic biocatalysts. We previously reported that light illumination enhances the chemical and optical yields in the cyanobacteria-catalyzed reduction of exogenous ketones (Nakamura and Yamanaka 2002a, b). To investigate the effect of light on chemical yields of cyanobacterial ketone reduction precisely, an acetophenone derivative, α,α,α-trifluoroacetophenone (TFA) was selected as the substrate for bioconversion because the ketone was easily reduced by the cyanobacterium and the optical yields of the product alcohol were unchanged by light conditions. Havel et al. (2007) reported that cyanobacterial ketone reduction was enhanced by light or glucose. Takemura et al. (2009) indicated that enantioselectivities of the reduction by a cyanobacterium could be influenced by deletion of genes from an endogenous dehydrogenase that requires coenzymes such as NADH/NADPH, which provide reducing power. Moreover, Hölsch et al. identified the enzyme involved in the cyanobacterial reduction reaction and the enzymes are NADPH-dependent (Hölsch et al. 2008, Hölsch and Weuster-Botz, 2010). We propose that the reducing power generated by increasing concentrations of NADPH through photosynthetic electron transport can be used in the reduction of exogenous ketones to yield useful alcohols. In cyanobacteria, the concentration of NADPH under illuminated conditions is approximately 6.5-fold higher than that of NADH (Tamoi et al. 2005).

In this report, we investigate the process and mechanism of reduction of exogenous ketones in cyanobacteria (*Synechococcus *PCC 7942) from the point view of reducing power, in terms of endogenous NADPH produced through photosynthesis. Here, we show that NADPH produced through photosynthesis is definitely used in the reduction of exogenous ketones in cyanobacteria.

## Materials and methods

### Organism and culture

*Synechococcus elongatus *PCC 7942 (*Synechococcus*) was obtained from the Pasteur Culture Collection of Cyanobacteria (PCC) of the Institut Pasteur, France. The cyanobacterium was grown in BG-11 medium (Rippka 1988) under continuous illumination (13.4 μmol photons m^-2 ^·s^-1^) provided by fluorescent lamps (day-light type, 20 W; Toshiba) at 25°C on a rotary shaker. Cell density was estimated by the turbidity at 720 nm of the cell suspension using a spectrophotometer (U-3210; Hitachi, Japan).

### Chemicals

TFA (α, α, α-trifluoroacetophenone), PF (2', 3', 4', 5', 6'-pentafluoroacetophenone), and IAM (iodoacetamide) were purchased from Tokyo Chemical Industry (Tokyo, Japan). DCMU (3-(3,4-dichlorophenyl)-1,1-dimethylurea), D,L-glyceraldehyde, 5'-AMP, NADPH, and NADH were purchased from Nacalai Tesque (Kyoto, Japan). DBMIB (2,5-dibromo-3-methyl-6-isopropyl-*p*-benzoquinone) and IAA (iodoacetic acid) were purchased from Sigma-Aldrich (St. Louis, MO, USA). All of the reagents used were of analytical grade.

### Gas chromatography

The chemical yields for ketone reduction were determined by GC (gas chromatography) analyses using a Shimadzu gas chromatography system GC-14B with C-R6A equipped with a chiral GC-column (CP-cyclodextrin-B-2,3,6-M-19 [CPCD]; 25 m; He 2 mL/min). Naphthalene was used as an internal standard.

### Assay for cellular reduction of ketones

The ketones (2 mg) were added to cell suspensions of *Synechococcus *(turbidity at 720 nm was about 1.0; equivalent to 120-170 mg wet-weight) in BG-11 medium (20 mL) at the beginning of the incubation period. The reaction mixture was shaken at 140 rpm and 25°C under continuous illumination with a fluorescent lamp (daylight type, 0-53.6 μmol photons m^-2^·s^-1^). Reaction times ranged from 12 h to 84 h. The resulting mixture was extracted with ether and eluted from an Extrelut column (Merck, Darmstadt, Germany).

### Measurement of photosynthetic activity

Photosynthetic O_2 _evolution of *Synechococcus *was measured with a Clark-type oxygen electrode (Model 4004; Yellow Springs Instruments, USA) (Murakami and Fujita 1988). An O_2 _electrode was placed in a plastic chamber (volume; ca 3 mL) and maintained at 25 ± 0.1°C with circulating thermostated water. The cell suspension of *Synechococcus *was stirred at a continuous speed with a magnetic stirrer. The rate of O_2 _evolution before and after the addition of DCMU (to final concentrations of 0, 0.1, 1, 10, or 100 μM) in DMSO (dimethyl sulfoxide) (100 μL) was measured under illumination with a halogen lamp (12.5 μmol photons m^-2^·s^-1^).

### Assay for reduction of ketones with cell-free extracts

Acetone-dried powder was used as cell-free extract. Cell suspension of *Synechococcus *(ca. 100 mL) was centrifuged for 15 min at 8,000 rpm and 4°C. The precipitate was immediately treated with cold acetone (-20°C) and dried under vacuum overnight, which gave 40 mg of acetone-dried powder.

A reaction mixture (2 mL of 0.1 M potassium phosphate buffer, pH 7) containing 10 mg acetone dried powder, 2 mg substrate (TFA or PF), and 5 mg reductant (NADPH or NADH) were incubated for 44 h at 25°C in the dark. The resulting mixture was extracted with ether and eluted with an Extrelut column.

## Results

### Light-dependency of the reduction of exogenous ketones by cyanobacterial cells

**Figure 1:**
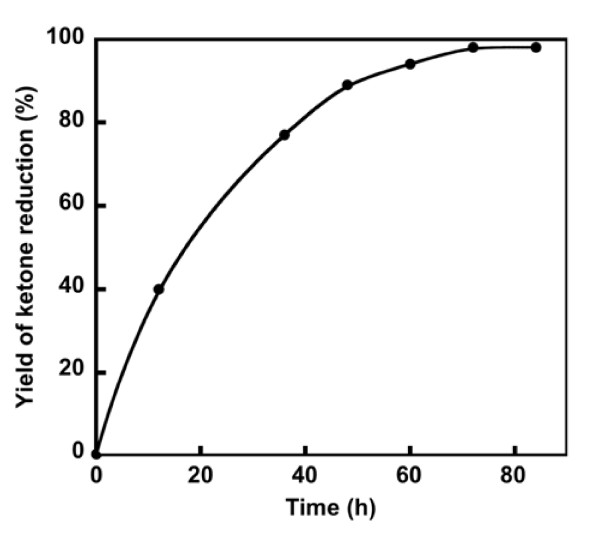
**Time course of the reduction of TFA by *Synechococcus***. TFA: 2 mg, Cell suspension of *Synechococcus*: 20 mL, 13.4 μmol photons m^-2^·s^-1^, 25°C.

A time-course of the reduction of a ketone (TFA) by *Synechococcus *under continuous illumination (13.4 μmol photons m^-2^·s^-1^) is shown in Figure 1. The reaction proceeded gradually and upon saturation reached a plateau after about 60 h. Therefore, the following experiments in this study were carried out with a 12-h incubation period.

**Figure 2:**
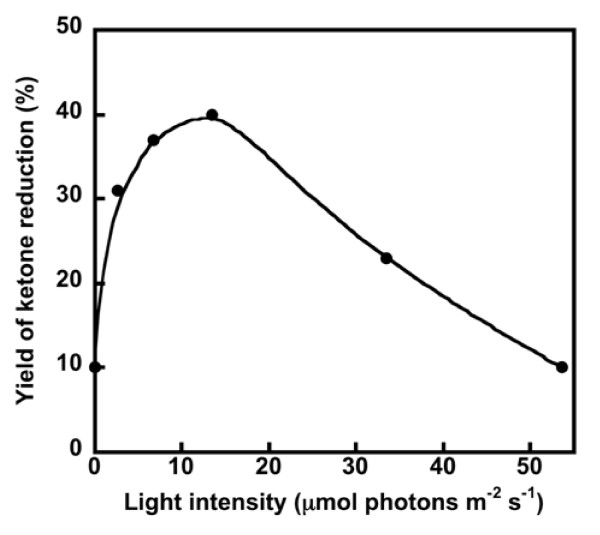
**Dependence of light intensity on the reduction of TFA by *Synechococcus***. TFA: 2 mg, Cell suspension of *Synechococcus*: 20 mL, 25°C, 12 h.

To investigate the dependency of light intensity on cyanobacterial ketone reduction in detail, the reduction activity was measured under varying light intensity (0, 2.7, 6.7, 13.4, 33.5, or 53.6 μmol photons m^-2^·s^-1^). As shown in Figure 2, the highest yield of TFA reduction was obtained in the reaction with 13.4 μmol photons m^-2^·s^-1^. In the case of the reduction of PF, the maximal reduction activity was also obtained at the same light intensity (data not shown). This light intensity might be compatible with the optimal light intensity for growth of *Synechococcus *under our experimental conditions. The enantiomeric excess of the reduction of TFA was 85% (*R*) and was found to be independent of the light intensity.

### Suppression of cyanobacterial ketone reduction by inhibition of photosynthetic electron transport

DCMU is a potent and specific inhibitor of photosynthetic electron transport (Fujita et al. 1987). TFA was incubated in an intact cell suspension of *Synechococcus *for 12 h under illumination (13.4 μmol photons m^-2^·s^-1^) with addition of DCMU (0, 0.1, 1, or 10 μM). Photosynthetic activity of *Synechococcus *was measured under the same light iintensity. The photosynthetic activity of *Synechococcus *was barely inhibited in the presence of 10 μM of DCMU and ketone reduction was suppressed depending upon concentrations of DCMU (Table 1). On the other hand, ketone reduction in the dark was found to be independent of the DCMU concentration (data not shown). The results indicate that cyanobacterial reduction of exogenous ketone is closely associated with photosynthetic electron transfer activity.

**Table 1:** Suppression of ketone reduction in *Synechococcus *by inhibition of photosynthetic electron transport

DCMU (μM)	Photosynthetic activity (relative)	Yield of ketone reduction (%)
0	1.0	43
0.1	0.42	44
1	0.16	36
10	0.038	15

TFA: 2 mg, Cell suspension of *Synechococcus*: 20 mL, 13.4 μmol photons m^-2^·s^-1^, 25°C, 12 h

DCMU was added at the beginning of the incubation period.

### Ketone reduction depends upon photosynthetic activity

**Figure 3:**
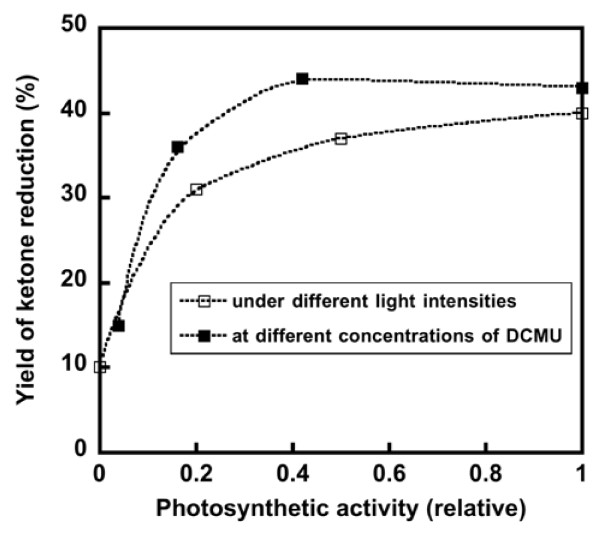
**Correlation of photosynthetic activity and reduction of TFA**. The data are from Table 1 and Figure 2.

The current results suggest that ketone reduction is associated with photosynthetic activity. The relationship between yields of cyanobacterial TFA reduction and photosynthetic activity was investigated under varying light intensity or under different concentrations of DCMU. The data from Figure 2 and Table 1 are plotted together in Figure 3. Thus, the result shows that cyanobacterial ketone reduction depends upon the photosynthetic activity of the cyanobacteria.

### Enhancement of ketone reduction by suppression of NADPH consumption in the Calvin cycle

**Figure 4:**
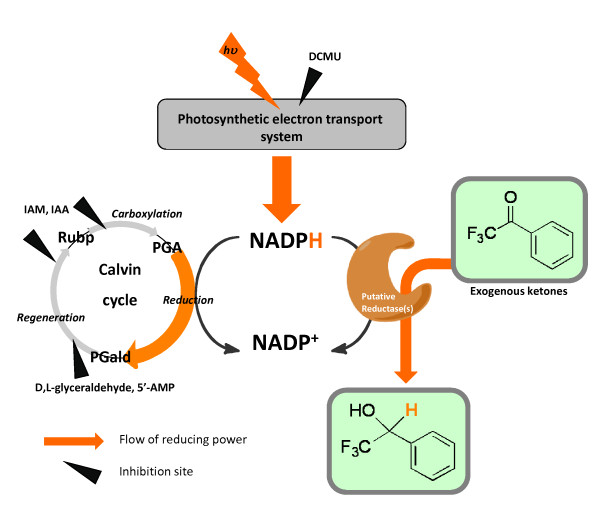
**Proposed scheme of the reduction of exogenous ketones by photosynthetically generated NADPH**. PGA: phosphoglycerate, PGald: phosphoglyceraldehyde, RuBp: ribulose-1,5-bisphosphate.

In the reduction step of the Calvin cycle, a large amount of NADPH (6 equivalents of NADPH per cycle) generated photosynthetically was found to be consumed (Figure 4). Therefore, we used inhibitors of the Calvin cycle to examine the involvement of NADPH in cyanobacterial ketone reduction. Ribulose-1,5-bisphosphate carboxylase-oxygenase (RuBisCo) catalyzes the carboxylation step and ribulose-5-phosphate (Ru5P) kinase catalyzes the regeneration step, both of which are reactions of the Calvin cycle (Wirtz et al. 1982). These enzymes are inhibited by SH-reagents such as iodoacetamide (IAM) or iodoacetic acid (IAA) (Figure 4). IAM or IAA (0.1 or 1 mM) was added in cell suspensions of *Synechococcus *with TFA under illumination. As shown in Table 2, IAM and IAA both enhance the reduction of TFA by *Synechococcus *(1.5-fold increase in the yield of reduction).

**Table 2:** Enhancement of ketone reduction in *Synechococcus *by inhibition of the Calvin cycle

	Inhibitor	Concentration (mM)	Yield of ketone reduction (%)
Experiment 1	None	-	34 (1.00)*
	IAM	0.1	43 (1.26)*
	IAM	1	52 (1.53)*
	IAA	0.1	39 (1.15)*
	IAA	1	61 (1.49)*

Experiment 2	None	-	40 (1.00)*
	D,L-glyceraldehyde	10	57 (1.43)*
	5'-AMP	5	59 (1.48)*

TFA: 2 mg, Cell suspension of *Synechococcus*: 20 mL, 13.4 μmol photons m^-2^·s^-1^, 25°C, 12 h

Inhibitors were added at the beginning of the incubation period.

*Values in parentheses are relative to the yield without inhibitor.

Moreover, the effects of the inhibitors (D,L-glyceraldehyde and 5'-AMP) of phosphoribulose kinase or isomerase which are involved in the process of regeneration of RuBp (ribulose-1,5-bisphosphate) (Forsee and Kahn 1972, Slabas and Walker 1976) were investigated. As shown in Table 2, the yields of the reduction of TFA by the cyanobacterium were increased by the addition of D,L-glyceraldehyde or 5'-AMP (1.5-fold increase in the yield of reduction).

These results surely suggest that inhibition of the Calvin cycle increases the levels of NADPH, which can then be used to reduce an exogenous ketone.

### Ketone reduction by dark respiratory activity of cyanobacteria

The TFA reduction by *Synechococcus *proceeded to some extent even in the dark (Figure 2) and DCMU did not completely suppress the reaction (Table 1). In cyanobacteria, the respiratory electron transport system is shared with the thylakoidal photosynthetic system and produces NADPH in the dark. Addition of DBMIB, an inhibitor of Cyt *b_6_-f*, resulted in cessation of TFA reduction in the dark, although the chemical yield of ketone reduction without DBMIB was 16% (data not shown). These results suggest that the reducing power with respect to exogenous ketones is also supplied by the respiratory system, even though the supply of reductants is limited.

### Confirmation of coenzyme dependency on cyanobacterial ketone reduction using a cell-free system

The current results suggest that endogenous reductase(s) is involved in cyanobacterial ketone reduction. To characterize the reductase(s), we investigated the ketone reduction activity of both the soluble and insoluble fractions. More than 95% of the reduction activity was detected in the soluble fraction (data not shown). This suggests that one or more hydrophilic enzymes participate in the artificial ketone reduction reaction. We confirmed the coenzyme (NADH/NADPH) dependency of the putative reductase(s) using the soluble fraction prepared from the cyanobacterium as an acetone-dried powder. It was found that NADPH is preferentially used in the reduction for both TFA and PF (Table 3). The results demonstrate that cyanobacterial ketone reduction is highly dependent on NADPH.

**Table 3:** Coenzyme (NADH/NADPH) dependency on ketone reduction using cell-free extracts of *Synechococcus*

Substrate	Coenzyme	Yield of ketone reduction (%)
TFA	NADPH	16 (1.00)*
	NADH	3.5 (0.22)*
		
PF	NADPH	12 (1.00)*
	NADH	0.8 (0.07)*

Substrate: 2 mg, 0.1 M potassium phosphate buffer (pH7): 2 mL,

Acetone-dried powder of *Synechococcus*: 10 mg, NADH or NADPH: 5 mg, 25°C, 44 h

*Values in parentheses are the relative to the yields in the presence of NADPH.

## Discussion

In this study, we demonstrated that the reduction of exogenously added ketones in cyanobacterial cells is highly dependent upon photosynthetic activity (Figure 2 and Table 1). Similar correlation curves for the reduction activities of ketones and the photosynthetic activities of the cyanobacterium were obtained from two different modulation mechanisms of photosynthetic activity (Figure 3). Since cyanobacterial ketone reduction was expectedly found to be enhanced by inhibitors of the Calvin cycle (Table 2), we propose that the reducing power of NADPH generated through photosynthesis (which is primarily used in CO_2 _fixation), can also be used for the reduction of exogenous ketones to produce the corresponding alcohols.

Reduction of substrates usually requires large amounts of energy. For the majority of redox enzymes, NADH and its phosphorylated form NADPH are essential endogenous coenzymes (Nakamura and Matsuda, 2002). As shown in Table 3, we confirmed that the ketone reduction observed in the present study is mediated by NADPH coenzyme. Coenzymes are prohibitively expensive if used in stoichiometric amounts. Since it is only the oxidation state of the coenzyme that changes during the reaction and in the reduction of a ketone oxidized form of the coenzyme generated, it may be regenerated *in situ *by using a second redox-reaction to allow it to re-enter the reaction cycle. Usually, formate (Seelbach et al. 1996), glucose (Wong et al. 1985), ethanol (Lam et al. 1988) and 2-propanol (Matsuda et al. 2000) are used to regenerate the oxidized form to the reduced form. These coenzymes are originally derived from photosynthetic products. In *Synechococcus*, glucose has also been used as a supplier of NADPH for ketone reduction (Havel et al. 2007). The previous research is compatible with the present results in terms of using NADPH as a reductant. The addition of DCMU did not lead to complete inhibition of cyanobacterial ketone reduction. In fact, cyanobacteria generally produce NADPH mainly through the photosynthetic reaction. Secondary sources of NADPH include the pentose phosphate cycle system in respiration. NADH is additionally produced through glycolysis (Scherer et al. 1988) as the major reductant. Indeed, the addition of DBMIB, a respiratory inhibitor, was found to reduce cyanobacterial ketone reduction activity (data not shown).

As shown in Figure 2, the reduction activity under illuminated conditions was found to be 4-fold higher than the reduction activity in the dark. Tamoi et al. reported that the NADPH/NADH ratio under light conditions is higher than the reduction activity in the dark, although the difference is less than 4-fold in the cells of *Synechococcus *PCC 7942 (Tamoi et al. 2005). We previously reported that light conditions change the enantiomeric excess of the products of the cyanobacterial reduction (Nakamura and Yamanaka, 2002b). In the ketone reduction reaction catalyzed by plant cultured-cells, different stereoselectivities have been obtained under light conditions with high CO_2 _concentrations and under dark condition with glucose (Kojima et al. 2009). Since the selectivity of the enzymatic reaction is fairly high in most cases, the low enantioselectivity usually means that a plurality of enzymes is involved in the reaction. In the present research, we have determined that light may increase the number of activated enzymes involved in the reduction in addition to increasing the concentration of NADPH. Hölsch et al. identified the enzymes involved in the cyanobacterial reduction reaction (Hölsch et al. 2008, Hölsch and Weuster-Botz, 2010). The reductases from Synechococcus PCC 7942 reduced acetophenone derivatives and may involve in our cyanobacterial ketone reduction. However, there may be some additional enzymes involved in the cyanobacterial TFA reduction because of the low enantioselectivity.

In summary, we have illustrated a possible mechanism for the reduction of exogenous ketones in cyanobacterial cells (see Figure 4). Exogenously-added ketones could be reduced by NADPH generated photosynthetically and NADPH is consumed in competition between the enzymes of the Calvin cycle and putative ketone reductases. Consequently, we have developed a new and useful application of cyanobacteria. The appropriation of NADPH generated by photosynthesis appears to contribute to a conversion mechanism for inexhaustible solar energy. The application of photosynthetic microalgae as biocatalysts using solar energy is expected to be developed in various efforts employing biotechnology, electrochemistry, and plant physiology (Abed et al. 2009). It is expected that photosynthetic microalgae will contribute to the development of many industrial synthetic reactions for production of useful compounds as well as efforts to design and optimize bioreactors (Walker et al. 2005) and to remediate environmental pollutants (Kuritz and Wolk 1995, Dubey SK et al. 2011).

## Abbreviations

DBMIB: 2,5-dibromo-3-methyl-6-isopropyl-*p*-benzoquinone (dibromothymoquinone); DCMU: 3-(3,4-dichlorophenyl)-1,1-dimethylurea; DMSO: dimethyl sulfoxide; GC: gas chromatography; IAA: iodoacetic acid; IAM: iodoacetamide; NAD(P)H: nicotinamide adenine dinucleotide (phosphate); PF: 2', 3', 4', 5', 6'-pentafluoroacetophenone; TFA: α, α, α-trifluoroacetophenone

## Competing interests

The authors declare that they have no competing interests.
